# The Courtship Behavior and the Ultrastructure of Sex Pheromone Glands in the Hind Tibiae of Male Ghost Moth *Endoclita davidi* (Lepidoptera: Hepialidae)

**DOI:** 10.3390/insects17010045

**Published:** 2025-12-30

**Authors:** Xingrui Huang, Shan Chen, Xing Li, Zihao Zhou, Qiong Zhou

**Affiliations:** College of Life Sciences, Hunan Normal University, Changsha 410081, China; huangxingrui@hunnu.edu.cn (X.H.); zihao_zhou@aliyun.com (Z.Z.)

**Keywords:** *Endoclita davidi*, courtship behavior, ultrastructure of sex pheromone glands, scent scale

## Abstract

The courtship behavior of moths is closely related to the biosynthesis and release of sex pheromones. To understand the mechanisms of the reproductive behavior of the ghost moth *Endoclita davidi*, a host insect of *Cordyceps xuefeng* (*Ophiocordyceps xuefengensis*), this study examined the courtship behavior and the ultrastructure of the sex pheromone gland of *E. davidi*. Infrared photography was used to analyze the courtship behavior, while SEM, paraffin tissue sectioning, and TEM were employed to examine the sex pheromone glands on the hind tibia. The courtship behavior of *E. davidi* occurs during the scotophase, during which males vibrate their wings and unfold the hairpencils located on the hind tibiae to lure the female moths. The hairpencil’s surface features regular longitudinal ridges interspersed with multiple circular pits. Multiple small holes were evident on the slightly enlarged areas close to the ends of the hairpencils. These small holes, formed at the base of the circular pits, connect to the internal glandular duct within the hairpencil, which links to the tibia glandular canal. The epidermal layer of the hind tibiae contains numerous glandular canals and dense microvilli. The glandular cells, specialized from dermal cells, contain numerous mitochondria, rough endoplasmic reticulum, clear vesicles of varying sizes, and round lipid droplets. The sex pheromone glands of male *E. davidi* release pheromones through the hairpencils in the hind tibia. The courtship behavior and ultrastructure of the sex pheromone gland of the male *E. davidi* provide a basis for further understanding pheromone release and communication mechanisms.

## Simple Summary

*Endoclita davidi* bores into and feeds on the stems and roots of *Clerodendrum cyrtophyllum*. The larvae, when parasitized by the fungus *Ophiocordyceps xuefengensis*, form valuable Xuefeng Cordyceps. To understand the mechanisms of *E. davidi*’s reproductive behavior, we studied its courtship behavior and sex pheromone glands. Infrared videos showed that the courtship behavior of *E. davidi* occurred during the scotophase, during which males vibrate their wings and unfold the hairpencils located on the hind tibiae (the third segment of the hind legs). The bottom of the pits in the bulging apical region of the hairpencils connects to the tibial glands; the epidermal cells of the hind tibiae develop into gland cells with dense microvilli. We confirmed that the male *E. davidi*’s pheromone glands are located in the hind tibiae and release pheromones via scent scales. Research on the ultrastructure of the sex pheromone glands in *E. davidi* provides a basis for further elucidating the mechanisms underlying sex pheromone release and sexual information communication.

## 1. Introduction

*Endoclita davidi* (Synonym *Phassue giganodus*), primarily a stem and root borer of *Clerodendrum cyrtophyllum* (Tubiflorae: Verbenaceae), is mainly distributed in Guangxi, Fujian, and Hunan Provinces in China [1,2,3]. The larvae of *E. davidi* are infected by *Ophiocordyceps xuefengensis*, an entomogenous fungus belonging to Ascomycota, leading to the formation of Xuefeng Cordyceps. This rare Chinese herbal medicine exhibits pharmacological activities analogous to those of Cordyceps sinensis [4,5,6]. Currently, research on *E. davidi* primarily focuses on the molecular identification of different insect forms [7], the characterization of antennal sensillum types [8], the nutritional composition analysis of larvae [9], and investigations into the reproductive behavioral rhythm of adults [3].

The courtship behavior in insects is a crucial aspect of the reproductive process, typically involving a sequence of complex visual, auditory, and chemical signal exchanges. Among these signals, sex pheromones serve as key chemical mediators in insect courtship [10]. Sex pheromones are chemical substances secreted by specialized glands on the body surface and released into the external environment to elicit courtship behavior among heterosexual conspecifics [11,12]. The location and characteristics of these secretory glands differ among insect species. Lepidopteran insects have been extensively studied with respect to sex pheromones, with research focusing on the morphological characteristics of sex pheromone glands in Noctuidae [13,14,15,16], Pyralidae [17,18,19], and Geometridae [20,21]. For moths, sex pheromone glands are primarily located in the following regions:

(1) The eversible bladder type, composed of specialized glandular epithelial cells in the intersegmental membrane between the eighth and ninth abdominal segments at the posterior end of the female abdomen. Representative species include *Helicoverpa zea* [14] and *Trichoplusia ni* [13] (Noctuidae), *Conopomorpha sinensis* (Gracillariidae) [22], *Scopula subpunctaria* (Geometridae) [23], and the female moths of *Hepialus deainensis* (Hepialidae) [24].

(2) The thoracic gland type, located on the dorsal or ventral thoracic surface. In Psychidae, three saddle-shaped glandular areas are present on the dorsal thorax. Additionally, the pheromone gland of female *Clania variegata* (Psychidae) is situated on the thoracic tergite [25], while in male *Ceromitia chalcocapna* (Adelidae), it is located on the posterior thoracic sternum [26].

(3) The specialized scale/pouch-like structure type, distributed on the wing margins, abdominal apex, or leg attachment sites. Representative examples include the hairpencils near the proximal margin of the costal fold on the forewings of male Tortricidae and Pyralidae moths [27], the wing-associated glands of male *Ephestia elutella* (Pyralidae) [28], the abdominal brushes at the abdominal apex of male *Grapholita molesta* (Tortricidae) [29], the tarsal segments of male *Helicoverpa zea*, *Helicoverpa armigera*, *H. assulta*, and *Heliothis virescens* (Noctuidae) [30], as well as the hind tibial brushes of male *Hepialus humuli* [31], *Hepialus hecta* [32], and *Endoclita signifer* (Hepialidae) [33]. However, the ultrastructural characteristics of the sex pheromone gland in *E. davidi* have not been reported to date. Building on our previous understanding of the reproductive behavioral rhythm of *E. davidi* [3], we further investigated the courtship behavior process of this moth and the ultrastructural features of the male sex pheromone gland. This study aims to provide a foundation for the accurate extraction, isolation, identification, and utilization of *E. davidi* sex pheromone, as well as to clarify the mechanism underlying its sexual chemical communication.

## 2. Materials and Methods

### 2.1. Insect Source and Rearing Conditions

The pupae and larvae of *E. davidi* were collected in September 2016, 2017, and 2019 from Dongkou County, Shaoyang City, Hunan Province (27°48′ N/110°30′ E, altitude 300–600 m), and then transported to the laboratory in the Life Science College of Hunan Normal University, China. The larvae were individually reared in tissue culture flasks (8.5 cm height; 6 cm diameter) with artificial feed (Table S1) within the Artificial Climate Chamber (RXZ-280D-LED; Ningbo Jiangnan Instrument Factory, Ningbo, China). The Chamber was maintained at 19–22 °C, 80 ± 2% RH with complete darkness. After pupa eclosion, healthy female and male moths with normal wing expansion were selected for testing.

### 2.2. Observation of Courtship Behavior

The female and male moths on the day of eclosion were collected and caged (30 cm × 30 cm × 30 cm) together with male and female in pairs, with 22 cages in total. The lab was kept at 25 ± 1 °C and 60 ± 5% relative humidity. The behavior of adults in cages was captured using an infrared camera (Rich HD-A210; Shenzhen Lenyin Technology Company, Shenzhen, China). The shooting time was determined to be 23:00 a.m.–03:00 a.m. the next day [3], according to the observation of the pre-experiment. After that, the courtship behavior procedure was recorded through the analysis of the video data.

### 2.3. The SEM Sample Preparation

For SEM (Scanning Electron Microscope), the hind tibiae were collected from 1-day-old male moths, and some of the scent scales above the tibiae were carefully removed. The samples were processed following the method described by Li et al. [8]: The prepared male hind tibiae were attached to sample stubs using conductive silver paste. These samples were then coated with gold using a vacuum sputtering device and observed under a JSM-6490LV SEM (JEOL Ltd., Tokyo, Japan) at an acceleration voltage of 20 kV.

### 2.4. Paraffin Histological Sections

The hind tibiae of freshly emerged male moths were fixed in Bouin’s solution (LMAI Bio; Shanghai, China) for 1 day and then rinsed repeatedly with phosphate-buffered saline (PBS, pH 7.2). The samples were dehydrated through a graded series of ethanol solutions (75%, 80%, 85%, 90%, 95%, and 100%) for 1–2 h each. The samples were then cleared in a mixture of absolute ethanol and xylene (1–2 h), followed by two clearings in pure xylene (0.5 h each). The samples were embedded in paraffin, sectioned longitudinally and transversely at a thickness of approximately 6 µm, stained with hematoxylin and eosin, and observed and photographed under an optical microscope (OLYMPUS BX51; Olympus Corporation, Tokyo, Japan).

### 2.5. TEM Sample Preparation

For TEM (Transmission Electron Microscopy), the hind tibiae and middle tibiae of 1-day-old male moths were fixed in 2.5% glutaraldehyde (prepared in PBS) for at least 2 h. They were rinsed three times with 0.1 M PBS for 10–15 min each (fixed with 1% osmic acid for 2 h, rinsed again three times with 0.1 M PBS, and dehydrated through a graded series of acetone solutions (50%, 70%, and 90%) for 10–15 min each, followed by dehydration in pure acetone for 15–20 min each). The samples were soaked in a mixture of acetone and embedding medium (1:1) at 37 °C for 12 h and then embedded in pure embedding medium for 10–12 h. They were oven-dried at 37 °C overnight and fixed at 60 °C for 12–24 h. Ultrathin sections (50–100 nm) were cut, stained with 3% uranyl acetate and lead nitrate, and observed and photographed under the HT7700 TEM (Hitachi, Tokyo, Japan).

## 3. Results

### 3.1. The Courtship Behavior of Endoclita davidi

**Figure 1:**
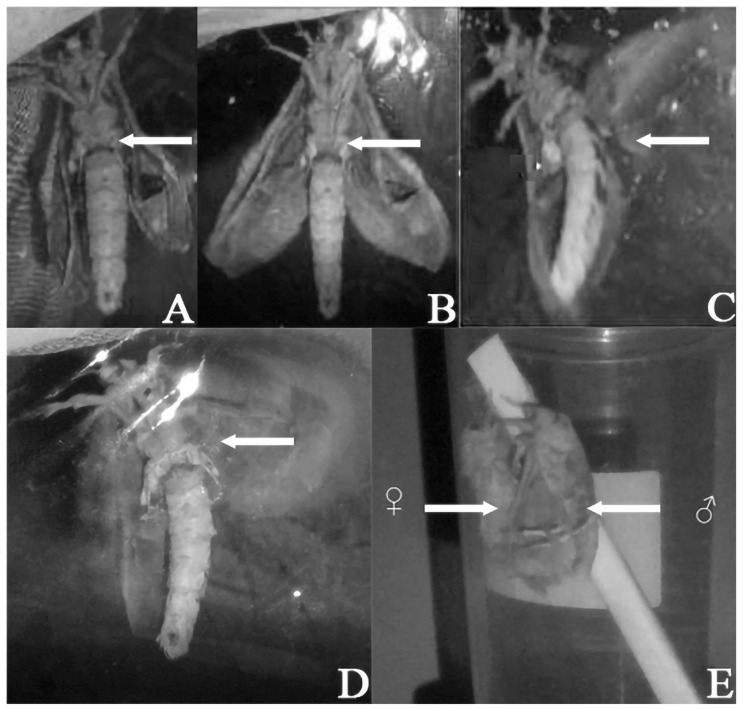
Calling behavior of male adult *Endoclita davidi*. (**A**) Male *E. davidi* keeps wings closed still at the sides of the body. (**B**) Male *E. davidi* is excited, flapping its wings in low frequency, and its hind tibia brushes have not yet opened. (**C**,**D**) Male *E. davidi* is in a highly excited state, wings flapping rapidly, and its hind tibia brushes opened. (**E**) Mating behavior of *E. davidi* (♀: female; ♂: male). Sc: Scent scale of hind foot tibia (indicated by the arrows).

Behavioral observations in the laboratory: During the daytime, male and female moths climb onto the top of the net cage using their forelegs and middle legs, remaining stationary with their wings covering both sides of their bodies. The female and male *E. davidi* moths in cages can engage in courtship and copulation on the evening of eclosion. According to the analysis of the video taken by the infrared camera, the courtship behavior of this moth is roughly shown as follows: The male moth that is hanging moves first, the antennae are raised, the two wings change from the state of being flat on both sides of the body during the day (Figure 1A) to the state of being immobile at an angle of about 45° with the body and begin to beat the wings (Figure 1B), and even twist the abdomen. After that, the female moth also changes from a static climbing state during the day to slightly unfolding her wings and slightly swinging her abdomen. Over time, the male moth’s wings beat at a higher frequency (Figure 1C) and gradually spread the smell brush of the hind tibiae, and the end of the abdomen begins to circle or swing left and right while climbing on the top of the cage and staying near the female moth. During this process, the female moth also begins to vibrate its wings, climb, and approach the male moth. Thus, both sides were in a state of excitement, flapping their wings with high frequency, and the abdomen twists strongly to try to make the ends of the abdomen touch each other (Figure 1D). When the abdominal ends of female and male moths are stably connected, mating behavior can be carried out (Figure 1E). A detailed description of the specific process of courtship behavior in *E. davidi* is provided in Table 1.

In the 22 cages observed, the male moths were active in most cases. There is only one special case: the female moth flaps its wings first, the end of its abdomen sprays out a liquid, and then the male moth flaps its wings and moves closer to the female moth.

### 3.2. SEM of the Hind Tibiae and Hairpencils of the Male E. davidi

**Figure 2:**
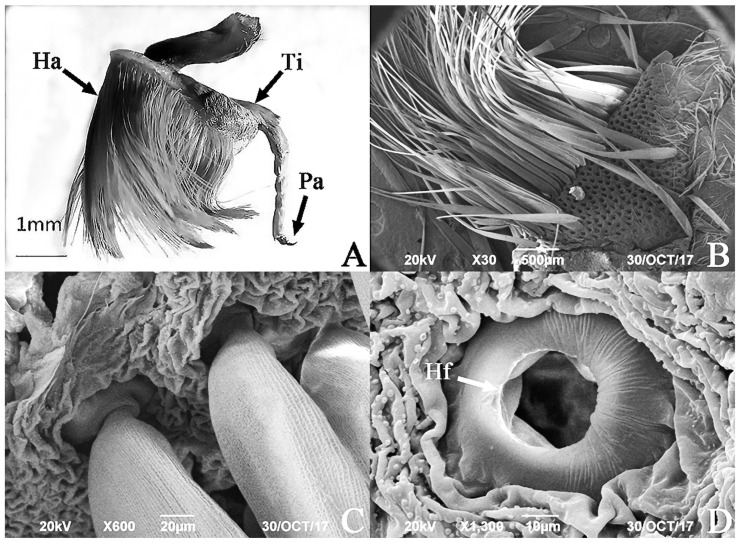
The SEM of the hind tibia of male *Endoclita davidi*. (**A**) Hind foot of the male. (**B**) Hind foot with removal partial hairpencil. (**C**) Surface of the hind tibia and base of the aromatic hairpencil. (**D**) Hair follicle at the base of the aromatic hairpencil. Note: Ha: hairpencil; Hf: hair follicle; Pa: paws; Ti: tibia.

The hind tibiae of the male *E. davidi* are slightly swollen into a pear shape, with a scent brush attached to its outer side (Figure 2A). The scent brush is composed of numerous golden yellow hair pencils. Hairpencils have a smooth surface, distributed on the entire outer side of the hind tibia. The basal half of each hairpencil is broader and more oblate (Figure 2B), with the widest point reaching 120.71 µm. The apical half is finer, with the thinnest diameter ranging from approximately 14.77 to 16.42 µm, averaging 15.53 ± 0.63 µm (*n* = 10).

The SEM revealed that the hairpencils are attached within specialized hair follicle fossae. The hair follicles are surrounded by a raised rim resembling a swimming ring. The outer diameter of the swimming ring averages 58.80 ± 5.16 µm (*n* = 10), and the inner diameter averages 29.37 ± 3.43 µm (*n* = 10). Each hair follicle houses a single hairpencil (Figure 2B,C), and the surface of the tibia between the follicles is wrinkled (Figure 2C). The hair follicle fossae of the hairpencils are connected to glandular ducts on the tibia (Figure 2D).

**Figure 3:**
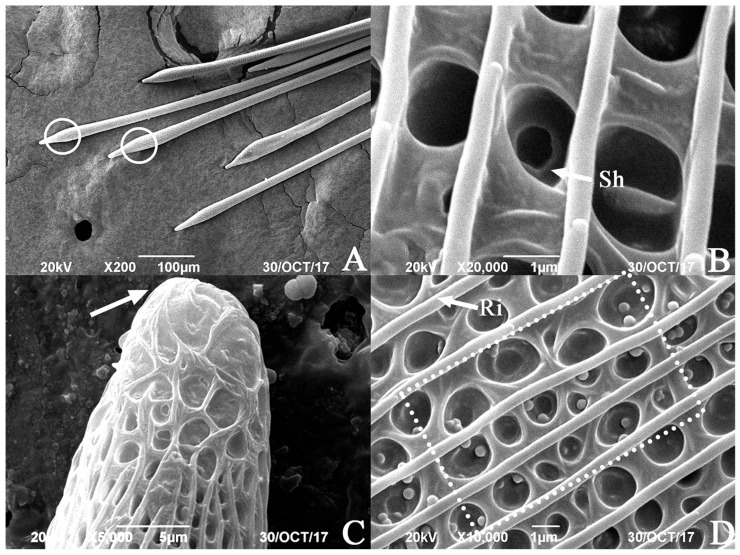
The SEM of male *Endoclita davidi* hind tibia scent scale. (**A**): Expansion position proximal of end of hairpencil (marked with white circles); (**B**): small hole in the concave pit (distributed near the slightly enlarged end); (**C**): near the base of whole grain (the end of the hairpencil is indicated by the arrows); and (**D**): the lateral wall of hairpencil is continuous with three granule belts (marked with white dashed line). Note: Sh: Small hole; Ri: Ridge.

The proximal end of the hairpencil is slightly intumescent, approximately 60 μm from its base, with a width of 24.61 ± 4.52 μm (*n* = 10) (Figure 3A). The bottom of the concave pits at the intumescent part has a distinct small hole leading into the internal glandular duct of the hairpencil (Figure 3B). The end of the hairpencil is blunt and nonporous (Figure 3C).

The surface of the hairpencil is adorned with neatly arranged longitudinal ridges (Figure 3D), with an average ridge width of 0.43 ± 0.028 μm (*n* = 10) and an average inter-ridge width of 1.58 ± 0.29 μm (*n* = 10). Between these ridges, there are uniformly distributed circular concave pits of varying sizes: the larger ones are more abundant and arranged in sequence, with diameters ranging from 1.40 to 2.05 μm and an average of 1.66 ± 0.22 μm (*n* = 10). Additionally, these larger pits have a secondary depression in the center, with diameters ranging from 0.48 to 0.81 μm and an average of 0.64 ± 0.12 μm (*n* = 10). The smaller pits have diameters ranging from 0.59 to 0.95 μm and an average of 0.68 ± 0.062 μm (*n* = 10), with no central depression. On the surface of the hairpencil, some spherical particles are arranged within the concave pits located between three adjacent longitudinal ridges that run the entire length (Figure 3D).

### 3.3. The Tissue Structure of Sex Pheromone Glands in the Hind Tibia of the Male E. davidi

**Figure 4:**
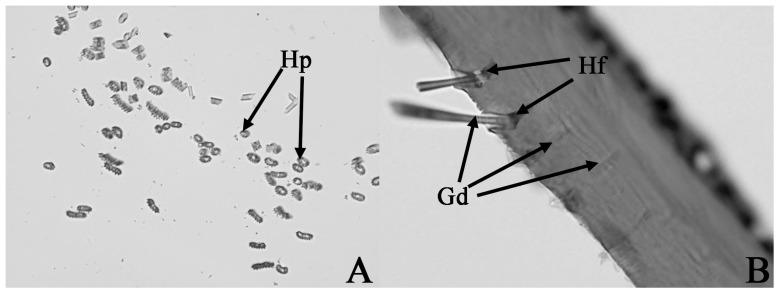
Hind tibiae tissue section of male *Endoclita davidi*. (**A**) Hind tibiae cross-sectional section diagram, denotes hollow hairpencil. (**B**) Hind tibia longitudinal section diagram, denotes the glandular ducts inside both the hairpencil and cuticle below the hair follicle. Note: Hp: hairpencil; Hf: hair follicle; Gd: glandular duct.

Tissue section observations of the hind tibiae of the male moths reveal that the hairpencils are a hollow tubular structure (Figure 4A), and there are glandular ducts beneath the base of the hair follicles. Below the glandular duct, it connects to gland cells in the epidermal layer through epidermal gland duct-connecting cells (Figure 4B).

### 3.4. The Ultrastructures of Sex Pheromone Glands in the Hind Tibia of the Male E. davidi

**Figure 5:**
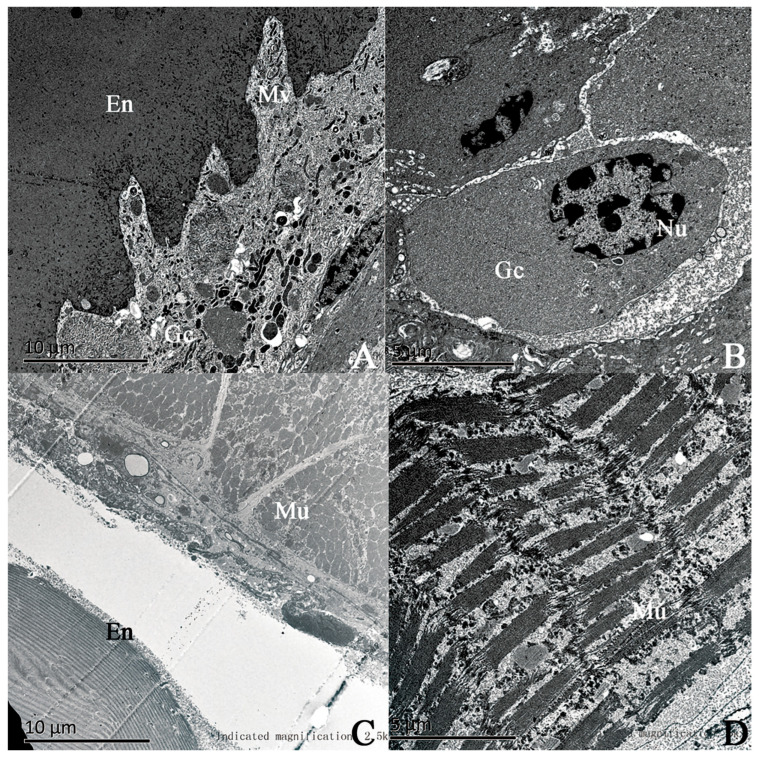
Comparison of the ultrastructure of the hind tibia (**A**) and the middle tibia (**B**) of male *Endoclita davidi* of 1d age. (**A**,**B**) Electron micrographs of the hind tibia. (**A**) denotes the microvilli of the gland cells under the epidermis. (**B**) denotes the gland cells. (**C**,**D**) Electron micrographs of the middle tibia. (**C**) denotes the cross-section of striated muscle. (**D**) denotes the longitudinal section of striated muscle. Note: En: endocuticle; Mv: microvilli; Mu: muscle; Gc: glandular cell; Nu: nucleus.

The results of TEM for the hind tibiae and middle tibiae of a male moth are shown in Figure 5. Comparing the ultrastructures of the two, it is observed that the epidermal cells underlying the tibial segment of the hind leg are specialized gland cells (Figure 5A,B). The microvilli at the tops of the gland cells are neatly and densely arranged and penetrate the inner epidermis, while there are numerous tiny pore canals distributed in the inner epidermis above the microvilli (Figure 5A). There are distinct intercellular spaces between the gland cells, and the nuclei are large and oval (Figure 5B). In contrast, the epidermal cells underlying the tibial segment of the middle leg are less developed, with developed striated muscle bundles on the inside (Figure 5C,D).

**Figure 6:**
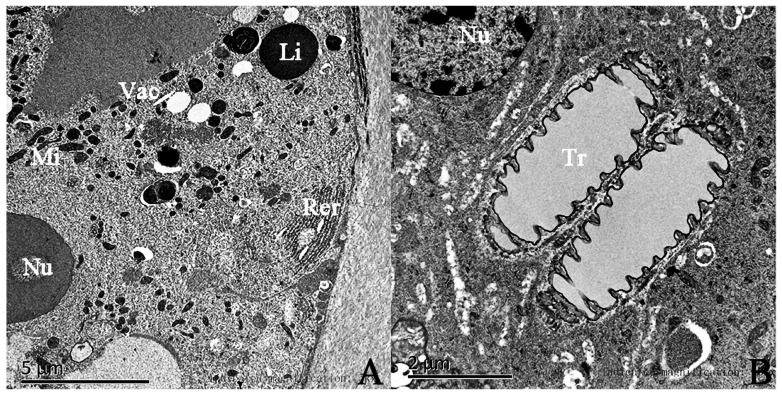
(**A**) The ultrastructure of the sex pheromone gland cells in the hind tibiae of male *Endoclita davidi*. (**B**) the tracheal structure in the cytoplasm of the glandular cells. Note: Vac: cavity; Mi: mitochondria; Nu: nucleus; Rer: rough endoplasmic reticulum; Li: lipid droplet; Tr: trachea.

Furthermore, the sex pheromone gland cells in the tibial segment of the male *E. davidi* possess many rod-shaped mitochondria. Within the cytoplasm, there is a well-developed rough endoplasmic reticulum accompanied by a large number of free ribosomes surrounding it, as well as transparent vesicles of varying sizes. These vesicles are often closely surrounded by numerous light gray round lipid droplets with relatively smooth surfaces. Additionally, there are structures such as tracheae present (Figure 6B).

## 4. Discussion

The courtship behavior of nocturnally active moths is strictly restricted to the scotophase. *Endoclita davidi* initiates courtship only in dark environments while remaining in a quiescent suspended state during the photophase (daylight or under indoor lighting). This behavior aligns with the general behavioral paradigm in moths, referred to as “photophase inhibition and scotophase activation,” thereby reinforcing the evolutionary adaptiveness of the scotophase as the optimal timing for moth courtship. Specifically, the scotophase reduces visual interference and lowers predation risk [34,35,36]. Furthermore, male *E. davidi* initiate wing vibration within 5 min after the start of the scotophase. This aligns with observations in other moth species: male diamondback moths (*Plutella xylostella*) reach the peak of courtship behavior immediately after light extinction [37], and the peak mating period of *Glyphodes caesalis* occurs within 0–3.5 h of the scotophase. These consistent responses reflect the high sensitivity of moths to scotophase initiation signals.

Under natural circumstances, the high-frequency wing-flapping exhibited by male moths likely involves circular flight patterns, which help in the effective dispersal of sex pheromones released by the hairpencil to attract females [38]. Notably, the wing-vibrating behaviors of male and female moths demonstrate distinct differences, consistent with the findings of *Endoclita excrescens* [39] and *Endoclita signifier* [33] within the Hepialidae family. In their natural habitat, male moths engage in group flights and initiate circling prior to female moths. Additionally, the flight behaviors of both sexes vary significantly, with the commencement of flight being closely associated with light intensity.

The surface ultrastructure of hairpencils exhibits certain conserved similarities among male moths of different species. For instance, male *Mythimna separata* [40] and *Endoclita vietnamensis* [41] possess hairpencils with hollow bristle shafts, as well as surface pores and longitudinal ridges—a structural feature consistent with the hairpencil morphology of *E. davidi*. SEM observations of the hind tibiae of male *E. davidi* revealed that the surfaces of their hind tibial hairpencils are characterized by regular longitudinal ridges and concave pits, with a species-specific distinction that some of these pits contain granular structures. These ultrastructural traits are inferred to constitute the physical basis for the golden yellow coloration of *E. davidi*’s hind tibial hairpencils. Notably, male *Hepialus humuli* [42] have been reported to utilize a combination of visual signals (from their silvery wings) and chemical signals for female attraction. Building on this finding, we hypothesize that male *E. davidi* may similarly employ the reflective properties associated with their golden yellow hairpencils to enhance female attraction during the scotophase. Alternatively, female *E. davidi* might be dually attracted during mating behavior: by olfactory cues from sex pheromones emitted by the male’s hind tibial hairpencils, and by visual stimulation derived from the golden yellow coloration of these structures.

Additionally, histological sections and SEM observations revealed that the hairpencil on the hind tibiae of male *E. davidi* is hollow in structure, with glandular ducts passing through the epidermal layer into the tibiae just below the base of the hair follicles. Each hairpencil is slightly swollen near its distal end, and numerous concave pits arranged regularly have small holes communicating with the lumen of the scales. Further TEM studies showed that the ultrastructural organization of the epidermal cells on the hind tibiae of male moths exhibits typical characteristics of sex pheromone-secreting gland cells. These cells are predominantly oval-shaped and clustered together, featuring numerous microvilli, large oval-shaped nuclei, and abundant organelles such as mitochondria, endoplasmic reticulum, and lipid droplets distributed within the gland cells. This structure is similar to that described by Percy and Macdonald [43]. Furthermore, Yang [44] and Hu [23] studied the ultrastructural organization of the sex pheromone glands in *Holcocerus vicarius* and the ultrastructural changes in sex pheromone glands at different developmental stages in *Scopula subpunctaria*, respectively. They found that not only the endoplasmic reticulum but also a significant number of lipid droplets are present within the gland cells. They suggested that lipid droplets serve as the raw material reservoir for sex pheromone production. Therefore, it can be inferred that the organelles within the gland cells of the hind tibiae of *E. davidi* are the sites for synthesizing sex pheromone precursors and storing crude products. Additionally, by comparing the structural features of the bundled striated muscles without gland cells in the mid tibiae of male *E. davidi*, along with the calling behavior and the unique structural characteristics of the hind tibiae of male moths, it can be speculated that during the initiation of courtship, the sex pheromone components are formed in the gland cells, pass through the glandular ducts in the epidermal layer of the tibiae, enter the intra-scale glandular ducts via the base of the hair follicles, and are ultimately released through the small holes in the concave pits between the longitudinal ridges near the distal end of the hairpencil. Furthermore, the wrinkled epidermal structure between the bases of the hairpencil of the hind tibiae in *E. davidi*, which resembles the layered folds and the absence of bristle coverage on the sex pheromone glands located on the intersegmental membranes between the eighth and ninth abdominal segments in *Conopomorpha sinensis* [22] and *Algedonia coclesalis* [17], is a retractable organ. During courtship in male *E. davidi*, the folds open, the hind tibiae expand, and the epidermis unfolds, allowing the hind tibia brushes to disperse, facilitating the full release of sex pheromones.

It is known that the majority of Lepidoptera release sex pheromones from females, such as *Heliothis virescens* [45], *Helicoverpa armigera* [16], *Helicoverpa zea* [14] of the Noctuidae, *Lambdina fiscellaria lugubrosa* [20] of the Geometridae, *Holomelina lamae* [46] and *Estigmene acrea* [47] of the Arctiidae, as well as *Deqin Hepialus* [24], *Korscheltellus gracilis* [48], and *Wiseana copularis* [49] of the Hepialidae. *Lycorea ceres* was the inaugural Lepidoptera species for which male pheromones were recorded and analyzed [50]. Subsequent studies have identified male pheromones in other Lepidoptera species, such as *Plodia interpunctella* [27] and *Ephestia elutella* [28] of the Pyralidae, *Choristoneura fumiferana* [51] of the Tortricidae, *Lycorea halia* [52] of the Nymphalidae, and *Hepialus hurnuli* [31] of the Hepialidae. The biological roles of volatiles released by male Lepidopteran hairpencils vary among species. For example, volatiles from the hairpencils of male *Grapholita molesta* exert a close-range attractant effect on females [53], while those from male *Pseudaletia separata* are proposed to inhibit the courtship responses of conspecific males towards females [54]. Building on the observed calling behavior of *E. davidi* and the principle of structural–functional adaptation [55], it can be reasonably inferred that the hind tibial hairpencils of male *E. davidi* function to release sex pheromones, thereby facilitating female attraction.

## 5. Conclusions

This study conducted a comprehensive analysis of the courtship behavior and sex pheromone gland ultrastructure of male *E. davidi*. Our results demonstrate that the courtship behavior of *E. davidi* occurs in the scotophase, during which males evert the hairpencils on their hind tibiae to lure female moths. Additionally, the hairpencil surface has regular longitudinal ridges with multiple circular pits, and small holes (formed at the pit bases) on its slightly enlarged tip areas connect to internal glandular ducts, which further link to tibia glandular canals; the hind tibia epidermal layer contains numerous glandular canals and dense microvilli, while dermal cell-specialized glandular cells harbor mitochondria, rough endoplasmic reticulum, vesicles, and lipid droplets. This work confirms that male *E. davidi*’s sex pheromone glands release pheromones via hind tibia hairpencils and provides a theoretical basis for further understanding lepidopteran pheromone release and communication mechanisms.

## Figures and Tables

**Table 1:** The sequence of calling and courtship behavior in *Endoclita davidi*.

Time	The Behavior of Male Adult *E. davidi*	The Behavior of Female Adult *E. davidi*
Within 5 min of entering the scotophase	The head of the hanging male moth begins to rush up and down, with its antennae gradually lifting from flat and close to its head to a more upright position. It starts flapping its wings, but the angle of wing-flapping is small (about 45°), indicating an excited state. The scent brush on the hind tibia (as indicated by the arrow in Figure 1A) has not yet been deployed.	From its resting state hanging on the top of the mesh cage, the female moth transitions into a position where its wings are slightly outstretched to the sides, forming an “eight” shape, without vibrating its wings. The abdomen slightly bends and swings both forward and backward, albeit at a small angle.
As the time goes on	Subsequently, the angle of the wings opening widens, and the flapping frequency increases, mimicking a flying posture. The male moth climbs towards the female on the cage top, and it can be observed that the scent brush on the hind tibia gradually unfolds and continuously vibrates. Meanwhile, the abdomen continuously draws circles or swings left and right, exhibiting a state of high excitement (Figure 1B).	The wings gradually begin to open at a small angle and vibrate as it climbs and approaches the male moth.
The scotophase lasts about 20 min	The male moth approaches the female, with its scent brush still unfolded, and its abdomen continues to swing left and right, adopting a mating posture. Simultaneously, it flaps its wings and uses its hind legs at a high frequency, displaying an extreme state of excitement (Figure 1C).	The female moth’s body begins to twist constantly, mainly manifested by intense bending and swinging of the abdominal tip in all directions. At the same time, she starts to flap her wings rapidly and intermittently at a high frequency. In two observed cases, before mating, the female moth ejected a stream of liquid from the abdominal tip, containing a few eggs.
The end of the calling	Both the male and female moths enter a stage of excitement and mutual approach, characterized by high-frequency wing flapping and abdominal twisting as they attempt to bring their abdominal tips into contact with each other (Figure 1D). At this point, the male moth approaches the female and hooks its legs onto the ventral side of the female’s chest. When the abdominal tips of the male and female moths come into contact, they enter the mating state (Figure 1E).	

## Data Availability

The original contributions presented in this study are included in the article/Supplementary Material. Further inquiries can be directed to the corresponding author.

## Supplementary Materials

The following supporting information can be downloaded at: https://www.mdpi.com/article/10.3390/insects17010045/s1, Figure S1: The ultrastructure of the epidermal cells in the hind tibiae of male *Endoclita davidi*. Ep: epidermis; Ec, endocuticle; Table S1: Artificial Feed Ingredient Details for *Endoclita davidi*.

## References

[1] Zhu H.F., Wang L.Y. (1985). On the stem-borers of Chinese Heplalids (Lepidoptera:Hepialidae). Acta Entomol. Sin..

[2] Wen T.C., Zhu R.C., Kang J.C., Huang M.H., Tan D.B., Ariyawansha H., Hyde K.D., Liu H. (2013). *Ophiocordyceps xuefengensis* sp. nov. from Larvae of *Phassus nodus* (Hepialidae) in Hunan Province, Southern China. Phytotaxa.

[3] Li X., Chen S., Zhou Q. (2021). Reproductive Behavior Rhythm of *Endoclita davidi* (Lepidoptera: Hepialidae), an Host Insect of *Ophiocordyceps xuefengensis*. J. Environ. Entomol..

[4] Fung S.Y., Cheong P.C.H., Tan N.H., Ng S.T., Tan C.S. (2018). Nutrient and Chemical Analysis of Fruiting Bodies of a Cultivar of the Chinese Caterpillar Mushroom, *Ophiocordyceps sinensis* (Ascomycetes). Int. J. Med. Mushrooms.

[5] Lu Z.H., Chen S.J. (2016). Review on the practice and perspective of the industrial production of *Ophiocordyceps sinensis*. J. Environ. Entomol..

[6] Wang Y., Zhang X.H., Zhang S.B., Wang X.L. (2020). Preliminary Study on Production and Antimicrobial Activity of Triterpenoidsfrom Xuefeng Congcao(*Ophiocordyceps xuefengensis*) by Liquid Cell Culture. China TCM Sci. Technol..

[7] Chen S., Zhou Q., Li G. (2017). DNA barcoding of various developmental stages of *Endoclita davidi* (Lepidoptera: Hepialidae) based on mtDNA COI gene sequence. Acta Entomol. Sin..

[8] Li G., Zhou Q., Chen S. (2017). SEM observation of antennal sensilla of adult *Endoclita davidi*, one host of *Ophiocordyceps xuefengensis*. J. Chin. Electron Microsc. Soc..

[9] Tang H.M., Fan Z.J., Zhou Q. (2020). Analysis and evaluation of nutrient components in wild and cultured *Endoclita davidi* larvae. J. Nat. Sci. Hunan Norm. Univ..

[10] Sánchez-Aros L., Queiroz A.F.O., Guajardo J., Barros-Parada W., Svensson G.P., Bergmann J. (2025). Characterization of a Novel Male Pheromone Compound in *Leucoptera sinuella* (Lepidoptera: Lyonetiidae) and Its Role in Courtship Behavior. Insects.

[11] Alam A., Abbas S., Abbas A., Abbas M., Hafeez F., Shakeel M., Xiao F., Zhao C.R. (2023). Emerging Trends in Insect Sex Pheromones and Traps for Sustainable Management of Key Agricultural Pests in Asia: Beyond Insecticides—A Comprehensive Review. Int. J. Trop. Insect Sci..

[12] Yang J.C., Zhang J.P., Wu C.Y., Bai Y., Guedes R.N.C., Dewer Y., Li F.-Q., Zang L.-S. (2025). Diversity and Role of Volatile Terpene and Terpenoid Pheromones in Insects. J. Econ. Entomol..

[13] Jefferson R.N., Shorey H.H., Gaston L.K. (1966). Sex Pheromones of Noctuid Moths. X. The Morphology and Histology of the Female Sex Pheromone Gland of *Trichoplusia ni* (Lepidoptera: Noctuidae). Ann. Entomol. Soc. Am..

[14] Raina A.K., Wergin W.P., Murphy C.A., Erbe E.F. (2000). Structural Organization of the Sex Pheromone Gland in *Helicoverpa zea* in Relation to Pheromone Production and Release. Arthropod Struct. Dev..

[15] Weatherston J., Percy J.E. (1968). Studies of Physiologically Active Arthropod Secretions: I Evidence for a Sex Pheromone in Female. Can. Entomol..

[16] Zhang S., Zhang Y., Ren S., Chen D. (1995). A Study on the Sex Pheromone-Producing Gland of the Cotton Bollworm *Helicoverpa armigera* Hübner (Lepidoptera: Noctuidae). Acta Entomol. Sin..

[17] Liu M., Yang M.F., Xu S.Y., Yao S.L. (2014). Calling Behavior of Adult *Algedonia coclesalis* (Lepidoptera: Pyralidae) and the Ultrastructure of the Sex Pheromone-Producing Glands in Its Female Adults. Acta Entomol. Sin..

[18] Tian Y., Liu M.Y. (1990). Location and ultrastructural of the sex pheromone glands in *Conogethes punctiferalis* Guenée. Acta Entomol. Sin..

[19] Zhang S.G., Zhang Y.H., Chen D.M. (1989). The morphology and structure of the sex pheromone glands of *Tryporyza intaca* Snellen and *Chilo infuscatellus* Snellen. Acta Entomol. Sin..

[20] Ostaff D.P., Shepherd R.F., Borden J.H. (1974). Sex Attraction and Courtship Behavior in Lambdina *Fiscellaria lugubrosa* (Lepidoptera: Geometridae). Can. Entomol..

[21] Ren Z.L., Zhao G., Xv J.H., Zhu H.Q. (1991). Biological studies on the sex pheromone of the sophora geometrid *Semiothisa cinerearia* Bremer et Grey. Acta Entomol. Sin..

[22] Zhang H., Chen X.Q., Wu G.Y., Jiang S.H. (2013). Observation for Sex-pheromone Gland Ultrastructure of Litchi Fruit Borer, *Conopomor pha sinensis*. Fujian J. Agric. Sci..

[23] Hu W.J., Chen W.L., Wei W. (2011). Comparative studies on ultrastructure of sex pheromone gland in female *Scopula subpunctaria* at different developmental stages. Chin. J. Appl. Entomol..

[24] Shen F., Yang Y.X., Shu C. (1991). Mating behavior and structure of secretory body of sex pheromone of Deqin chost moth (*Hepialus deqinensis*). Southwest China J. Agric. Sci..

[25] Zhao B.G. (1985). Studies on the Glands and the Release Mechanism of the Female Sex Pheromone in Clania Variegata Snell. J. Nanjing For. Univ. Sci. Ed..

[26] Wojtusiak J. (1999). A New Type of Scent Organ in Lepidoptera: A Giant Thoracic Pheromone-Disseminating Structure in a Male Incurvarioid Moth (Lepidoptera, Adelidae). J. Nat. Hist..

[27] Grant G.G. (1978). Morphology of the Presumed Male Pheromone Glands on the Forewings of Tortricid1 and Phycitid2 Moths. Ann. Entomol. Soc. Am..

[28] Phelan P.L., Silk P.J., Northcott C.J., Tan S.H., Baker T.C. (1986). Chemical Identification and Behavioral Characterization of Male Wing Pheromone of *Ephestia elutella* (Pyralidae). J. Chem. Ecol..

[29] Baker T.C., Cardé R.T. (1979). Courtship Behavior of the Oriental Fruit Moth (*Grapholitha molesta*): Experimental Analysis and Consideration of the Role of Sexual Selection in the Evolution of Courtship Pheromones in the Lepidoptera 2. Ann. Entomol. Soc. Am..

[30] Choi M.Y., Ahn S.J., Park K.C., Meer R.V., Cardé R.T.C., Jurenka R. (2016). Tarsi of Male Heliothine Moths Contain Aldehydes and Butyrate Esters as Potential Pheromone Components. J. Chem. Ecol..

[31] Mallet J. (1984). Sex Roles in the Ghost Moth *Hepialus humuli* (L.) and a Review of Mating in the Hepialidae (Lepidoptera). Zool. J. Linn. Soc..

[32] Schulz S., Francke W., König W.A., Schurig V., Mori K., Kittmann R., Schneider D. (1990). Male Pheromone of Swift Moth, *Hepialus hecta* L. (Lepidoptera: Hepialidae). J. Chem. Ecol..

[33] Chen X., Su X., Qiu Z., Xu Y., Yang Z., Hu P. (2024). Courtship and Mating Behavior of *Endoclita signifer* (Hepialidae: Lepidoptera) and the Male Sex Pheromones in Hairbrushes. J. Econ. Entomol..

[34] Sower L.L., Shorey H.H., Gaston L.K. (1970). Sex Pheromones of Noctuid Moths. XXI. Light: Dark Cycle Regulation and Light Inhibition of Sex Pheromone Release by Females of Trichoplusia Ni. Ann. Entomol. Soc. Am..

[35] Levi-Zada A., Byers J.A. (2021). Circadian Rhythms of Insect Pheromone Titer, Calling, Emission, and Response: A Review. Sci. Nat..

[36] van Geffen K.G., van Eck E., de Boer R.A., van Grunsven R.H.A., Salis L., Berendse F., Veenendaal E.M. (2015). Artificial Light at Night Inhibits Mating in a Geometrid Moth. Insect Conserv. Divers..

[37] Wang D., Yang G., Chen W. (2021). Diel and Circadian Patterns of Locomotor Activity in the Adults of Diamondback Moth (*Plutella xylostella*). Insects.

[38] Turner J.R.G. (2013). The Dawn Flight of the Gold Swift *Hepialus Hecta*: Predator Avoidance and the Integration of Complex Lek Behaviour (Lepidoptera, Hepialidae). Biol. J. Linn. Soc..

[39] Kan E., Kitajima H., Hidaka T., Nakashima T., Sato T. (2002). Dusk Mating Flight in the Swift Moth, *Endoclita excrescens* (Butler) (Lepidoptera: Hepialidae). Appl. Entomol. Zool..

[40] Birch M.C., Poppy G.M., Baker T.C. (1990). Scents and Eversible Scent Structures of Male Moths. Annu. Rev. Entomol..

[41] Liu J., Chen X., Hu P. (2024). Morphological and Histological Observations on the Hair Brush of *Endoclita vietnamensis* (Lepidoptera, Hepialidae). Microsc. Res. Tech..

[42] Andersson S., Rydell J., Svensson M.G.E. (1998). Light, Predation and the Lekking Behaviour of the Ghost Swift *Hepialus humuli* (L.) (Lepidoptera, Hepialidae). Proc. R. Soc. Lond. B Biol. Sci..

[43] Percy-Cunningham J.E.E., Macdonald J.A. (1987). Biology and Ultrastructure of Sex Pheromone–Producing Glands. Pheromone Biochemistry.

[44] Yang M.H., Zhang J.T., Fan L.H., Liu H.X., Luo Y.Q., Zong S.X., Cao C.J. (2011). Ultrastructural observation of the sex pheromone communication system in *Holcocerus vicarius* (Walker) (Lepidoptera:Cossidae). Acta Entomol. Sin..

[45] Hillier N.K., Vickers N.J. (2004). The Role of Heliothine Hairpencil Compounds in Female *Heliothis virescens* (Lepidoptera: Noctuidae) Behavior and Mate Acceptance. Chem. Senses.

[46] Yin L.R.S., Schal C., Cardé R.T. (1991). Sex Pheromone Gland of the Female Tiger Moth *Holomelina lamae* (Lepidoptera: Arctiidae). Can. J. Zool..

[47] MacFarlane J.H., Earle N.W. (1970). Morphology and Histology of the Female Sex Pheromone Gland of the Salt-Marsh Caterpillar, *Estigmene acrea*. Ann. Entomol. Soc. Am..

[48] Kuenen L.P.S., Wagner D.L., Wallner W.E., Cardé R.T. (1994). Female Sex Pheromone in *Korscheltellus gracilis* (Grote) (Lepidoptera: Hepialidae). Can. Entomol..

[49] Allan R.A., Wang Q. (2001). Mating Behaviour, and Evidence for a Female-released Sex Pheromone, in *Wiseana copularis* (Meyrick) (Lepidoptera: Hepialidae). N. Z. J. Zool..

[50] Meinwald J., Meinwald Y.C., Wheeler J.W., Eisner T., Brower L.P. (1966). Major Components in the Exocrine Secretion of a Male Butterfly (Lycorea). Science.

[51] Roscoe L.E., Silk P., Eveleigh E.S. (2016). Evidence of Male Hair Pencil Pheromone in *Choristoneura fumiferana* (Lepidoptera: Tortricidae). J. Insect Sci..

[52] Gnatzy W., Fischer O.W., Kiesel A., Vane-Wright R.I., Boppré M. (2020). Diverticula in Male *Lycorea halia* Butterflies (Lepidoptera: Nymphalidae: Danaini: Itunina)—Support Organs for Everted Hairpencils with Unique Ultrastructure. Neotrop. Entomol..

[53] Baker T.C., Nishida R., Roelofs W.L. (1981). Close-Range Attraction of Female Oriental Fruit Moths to Herbal Scent of Male Hairpencils. Science.

[54] Hirai K. (1982). Directional Flow of Male Scent Released by *Pseudaletia separata* Walker (Lepidoptera: Noctuidae) and Its Repellent Effect on Adults and Larvae of Four Noctuid and One Phycitine Moth. J. Chem. Ecol..

[55] Zhang J.T., Han Y., Gan Y.L., Meng X.Z. (2002). Location and histology of the sex pheromone-producing gland in *Holcocerus insularis*. Acta Entomol. Sin..

