# Supplementary material for: The Courtship Behavior and the Ultrastructure of Sex Pheromone Glands in the Hind Tibiae of Male Ghost Moth Endoclita davidi (Lepidoptera: Hepialidae)
Source: Insects. 2025 Dec 30;17(1):45. doi: 10.3390/insects17010045 (PMC12842401; doi:10.3390/insects17010045)
Supplement: Supplementary file 1 [file insects-17-00045-s001.zip › Figure S1.pdf]

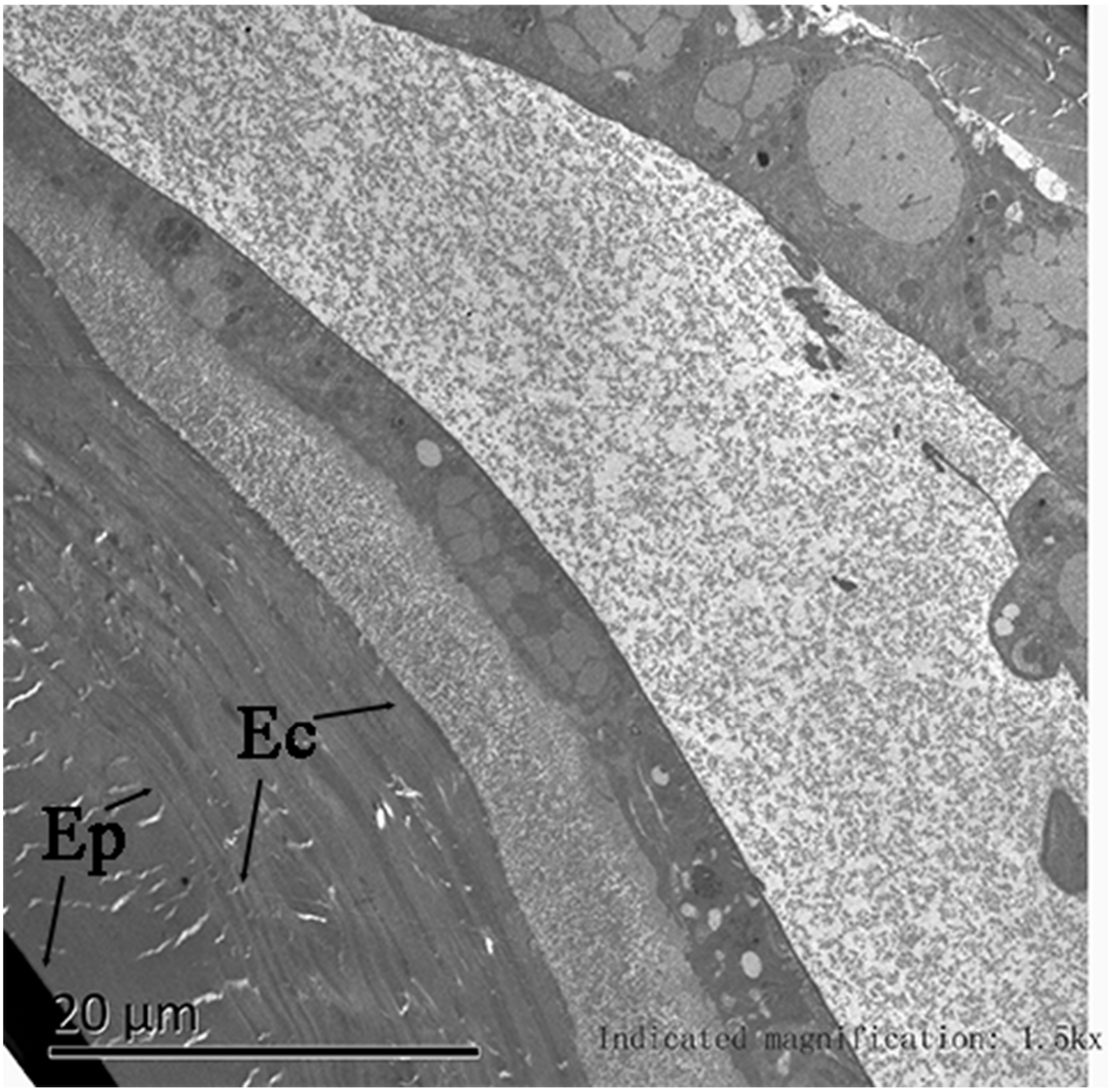

Figure S1. The ultrastructure of the epidermal cells in the hind tibiae of male *Endoclita davidi*.

Ep:epidermis; Ec, endocuticle.
