# Supplementary material for: The Courtship Behavior and the Ultrastructure of Sex Pheromone Glands in the Hind Tibiae of Male Ghost Moth Endoclita davidi (Lepidoptera: Hepialidae)
Source: Insects. 2025 Dec 30;17(1):45. doi: 10.3390/insects17010045 (PMC12842401; doi:10.3390/insects17010045)
Supplement: Supplementary file 1 [file insects-17-00045-s001.zip › Table S1. Artificial Feed Ingredient Details for Endoclita davidi.pdf]

Table S1. Artificial Feed Ingredient Details for *Endoclita davidi*

Feed Components	Dosage Range	Specific Requirements/Source
Indigowoad powder	20~40 g	Collected from the producing area of Hepialus armoricanus (root), dried naturally, then pulverized (passed through a 40~mesh sieve); stems/leaves can also be used, dried naturally or by baking then pulverized.
Carrot powder	0~30 g	Fresh carrots purchased, cut into small pieces with a diameter < 1 mm.
Soybean meal	50~70 g	Bulk products purchased from supermarkets or obtained by pulverizing dried soybeans.
Corn flour	30~50 g	Bulk products purchased from supermarkets or obtained by pulverizing dried corn.
Oatmeal/Wheat bran	20~40 g	Bulk products purchased from supermarkets.
Yeast	10~30 g	Types include yeast extract (this time using yeast extract from Beijing Aoboxing Biotechnology Co., Ltd.), yeast powder, round yeast, or dry yeast.
Vitamin	2~6 g	Types include vitamin C (this time using ascorbic acid), multivitamins, or 21 Golden Vitamins.
Cholesterol	2~4 g	Product from Shanghai Pharmaceutical Group.
Agar	30~50 g	Agar from BISHARP, Japan.
Water	900~1200 mL	Types include distilled water, tap water, mineral water, purified water, or double~distilled water.
Preservative	4~6 g	This time using sorbic acid; optional types include at least one of sorbic acid, benzoic acid, p~hydroxytoluene, sodium benzoate, potassium sorbate, calcium propionate, sodium propionate, methyl p~hydroxybenzoate, dehydroacetic acid, sodium diacetate, nitrate, nitrite, sodium lactate, sodium propionate, etc.
